# Survival Benefits of Second-line Axitinib Versus Everolimus After First Line Sunitinib Treatment in Metastatic Renal Cell Carcinoma

**DOI:** 10.1007/s12253-020-00809-z

**Published:** 2020-04-15

**Authors:** Lajos Géczi, György Bodoky, György Rokszin, Ibolya Fábián, László Torday

**Affiliations:** 1 Urogenital Tumors and Clinical Pharmacology Department, National Institute of Oncology, Ráth György u. 7–9, 1122 Budapest, Hungary; 2 Department of Oncology, Szent László Hospital, Albert Flórián út 5, 1097 Budapest, Hungary; 3 RxTarget Ltd, Bacsó Nándor út 10, 5000 Szolnok, Hungary; 4 University of Veterinary Medicine, István út 2, 1078 Budapest, Hungary; 5 Department of Oncotherapy, University of Szeged, Korányi fasor 12, 6720 Szeged, Hungary

**Keywords:** Metastatic renal cell carcinoma, Axitinib, Sequential therapy, VEGF inhibitors, Overall survival

## Abstract

**Background:**

Targeted therapies significantly improve clinical outcomes among patients with metastatic renal cell carcinoma (mRCC). Several new agents have been approved for first- and second-line use. However, there is a lack of compelling evidence comparing sequencing strategies, and available comparative data regarding the real-world effectiveness of different therapeutic sequences are limited.

**Materials and Methods:**

We identified mRCC patients who initiated targeted therapy between January 1, 2008 and May 31, 2017 from the National Health Insurance Fund (NHIF) database of Hungary. Overall survival (OS) and duration of first-line treatment (DFT) were obtained for patients receiving sunitinib-everolimus, sunitinib-axitinib, or pazopanib-everolimus treatment sequences. OS of sunitinib-everolimus and sunitinib-axitinib sequences was also determined for patients having better or worse response to sunitinib first-line therapy.

**Results:**

Median OS was significantly longer among patients treated with sunitinib-axitinib compared to those receiving sunitinib-everolimus. Median DFT was also significantly longer in the sunitinib-axitinib vs. sunitinib-everolimus group. Sunitinib-axitinib was associated with significantly longer median OS compared to sunitinib-everolimus in patients with better response to first-line sunitinib in the pooled sunitinib population. In patients with worse response to sunitinib, sunitinib-axitinib was associated with a trend towards greater OS compared to sunitinib-everolimus, but the difference did not reach statistical significance.

**Conclusions:**

In this nationwide database analysis, mRCC patients treated with the sunitinib-axitinib sequence had significantly longer OS compared to those receiving sunitinib-everolimus therapy. The OS benefits of second-line axitinib were consistent among patients with better response to sunitinib defined by DFT values.

## Introduction

Vascular endothelial growth factor (VEGF) and its receptors have a central role in the development and progression of renal cell cancer (RCC). The activation of VEGF-driven signal transduction pathways promotes tumor angiogenesis, growth and proliferation, as well as survival of malignant cells through the induction of expression of various anti-apoptotic factors [1, 2]. Targeting VEGF pathways either by VEGF ligand-binding blockade or by the inhibition of downstream signaling by VEGF receptor (VEGFR) inhibitors has become the mainstay of systemic treatment for metastatic RCC both in first and second line [3]. Eight of the 12 agents ever approved for the treatment of mRCC are anti-VEGFR agents (sorafenib, sunitinib, pazopanib, tivozanib, axitinib, cabozantinib, and lenvatinib), which inhibit tumor angiogenesis to varying degrees. These targeted therapies provide higher efficacy (in terms of PFS or OS) compared to the previous standard-of-care [4, 5].

Sunitinib is a highly potent, oral, multitargeted receptor tyrosine kinase inhibitor (TKI) which inhibits VEGFR-1, -2, and − 3, and a number of other receptor tyrosine kinases including platelet-derived growth factor receptor (PDGFR)-α and -β [6]. In its registration trial, sunitinib showed significant clinical benefits over IFN-α in the first-line treatment of mRCC patients and was approved for the treatment of mRCC in 2006. Sunitinib is recommended by international and national guidelines as one of the standard first-line treatment options for mRCC patients with good or intermediate prognosis based on the Memorial Sloan Kettering Cancer Center (MSKCC) criteria [7–10].

Despite the well-defined benefits of sunitinib, disease progression usually occurs after 6–15 months, and eventually sunitinib resistance develops in almost all patients. The mechanism of sunitinib resistance is thought to be multifactorial and is not yet fully understood [11–13]. Before the approval of nivolumab, cabozantinib and the lenvatinib-everolimus combination in 2018, options for second-line therapy for patients with advanced RCC who had progressed after initial VEGF-targeted systemic therapy included the VEGFR TKI axitinib [14] or the mammalian target of rapamycin inhibitor (mTORi) everolimus [15]. However, in the absence of head-to-head comparative second-line studies and validated biomarkers or clinical parameters which could predict the outcomes of a specific second-line therapy, clinicians are faced with the challenge of choosing between a growing number of available second-line agents. In the lack of proper guidance and validated data, treatment decisions often remain empiric and based on physician and patient preferences or toxicity profiles rather than high-quality evidence. Therefore, further studies are needed to optimize the sequence of targeted therapies for mRCC patients to provide further insights into the real-world effectiveness and safety profile of available regimens and treatment sequences. This challenge remained further exciting after the introduction of combined TKI and immunotherapy combination in first line setting.

Targeted therapies and their sequences were gradually incorporated into Hungarian clinical practice after their international approval for the treatment of mRCC. Sunitinib has been reimbursed in Hungary for the first -line treatment in good and intermediate risk of this disease since 2010, while pazopanib gained approval as first-line therapy in 2014. As per reimbursement rules, first-line pazopanib can only be followed by everolimus, which has been reimbursed since 2014 for second-line treatment after sunitinib or pazopanib. Axitinib has also been reimbursed since 2014 but only for second-line treatment after first-line sunitinib therapy.

The present analysis investigated OS and DFT among real-world mRCC patients receiving sunitinib-everolimus, sunitinib-axitinib, or pazopanib-everolimus sequential treatments in Hungary.

## Patients and Methods

This was a nationwide retrospective database analysis using prescription claims data from the database of the NHIF. The NHIF database contains prescription claims data of all reimbursed medicinal products from all Hungarian patients. Patients were selected for the research if they had at least one reimbursed prescription claim at a public pharmacy between January 1, 2008 and May 31, 2017 for any of the following drugs: IFN-α/IL-2, sorafenib, sunitinib, everolimus, axitinib, pazopanib, temsirolimus. The primary aim of our study was to determine the impact of approved targeted treatment sequences, namely, sunitinib-everolimus, sunitinib-axitinib, and pazopanib-everolimus on OS of patients with mRCC. We focused on patients treated these sequential targeted therapies where we could identify the exact date of first-line treatment initiation.

The analysis included patients who had the ICD-10 code C64 (‘Malignant neoplasm of kidney, except renal pelvis’). This identification process yielded a pool of social security numbers denoting mRCC patients who had received pharmacological treatment for mRCC during the examined period.

Since the NHIF database only contains reimbursed prescription claims data, non-reimbursed claims were not included in the analysis, and we did not have any information about the proportion of patients receiving drugs on the basis of individual import or in a clinical trial. The database does not contain laboratory data (e.g. glucose levels, hemoglobin A1c, lipids), patient parameters (e.g. body mass index), information on harmful addictions (e.g. alcohol or tobacco use), or prognostic features. Since we did not have any information on the baseline prognostic features of patients (e.g. TNM staging, MSKCC prognostic allocation or previous nephrectomy) receiving different sequential treatments, we used a reverse deduction method to adjust for this confounding. In order to create patient groups with similar response to first-line sunitinib, we pooled patients from the sunitinib-everolimus and sunitinib-axitinib groups and lined them up in a decreasing order of DFT. We divided them into two cohorts based on DFT in a way that both cohorts included the same number of patients. The so-called “upper 50%” cohort included patients who responded better to sunitinib (i.e. those with longer DFT, while those with poorer response (i.e. with shorter DFT, constituted the “lower 50%” cohort. The establishment of patient cohorts with similar baseline disease severity allowed us to perform informative comparisons between sunitinib-based sequential treatment groups.

The mathematical algorithm required for analysis was designed and provided by RxTarget Statistical Programming and Analysis Ltd. using SQL programming language based on a preliminary study plan, and was submitted via email to NHIF. Anonymized data were provided by NHIF in an aggregated form without any individual identification parameter based on a specific permit (NHIF approval number: S04/77/2017), with the strict consideration of data protection rules.

OS, defined as the time period between the initiation of first-line pharmacological therapy and death, and DFT, defined as the time period between the initiation of first-line pharmacological therapy and the initiation of second-line treatment were estimated using the Kaplan-Meier method and analyzed by log-rank test and Cox regression analysis. Overall statistical analysis was carried out using the R Software version 3.4.2 (09-28-2017) with the application of ‘survival’, ‘survminer’, ‘multicomp’, and ‘cowphw’ packages.

The Ethical Hungarian Medical Research Council approved the study (licence number: OGYÉI/31936-1/2017).

## Results

Between January 1 2008 and May 31 2017 318 patients were treated on sunitinib-everolimus (mean age: 60.9 years), 128 patients on sunitinib-axitinib (mean age: 62.1 years), and 66 patients on pazopanib-everolimus (mean age: 60.6 years) sequential therapy.

**Fig. 1:**
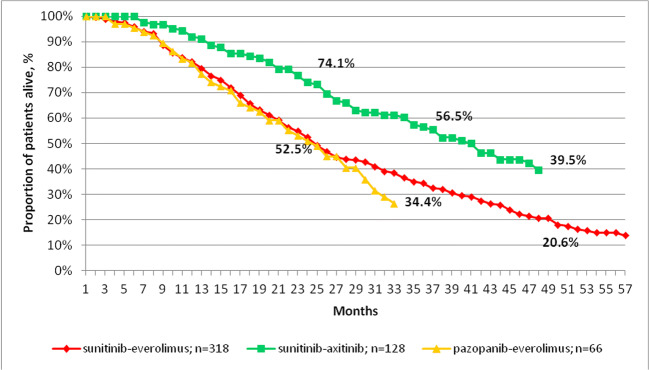
Overall survival from the initiation of first-line therapy among patients with mRCC receiving sunitinib-everolimus, sunitinib-axitinib, or pazopanib-everolimus sequential treatment

Figure 1 shows the OS of patients treated with sunitinib-everolimus, sunitinib-axitinib or pazopanib-everolimus starting from the initiation of first-line therapy. Patients with mRCC receiving sunitinib-axitinib had significantly longer median OS compared to those receiving the sunitinib-everolimus sequence (median OS: 41.0 vs. 21.7 months; p < 0.0001; HR: 0.55 [0.42–0.72]). Median OS of patients receiving the pazopanib-everolimus or sunitinib-everolimus sequences appeared to be similar. However, the pazopanib-everolimus sequence could not be directly compared to sunitinib-based sequences due to differences in first-line therapy.

**Fig. 2:**
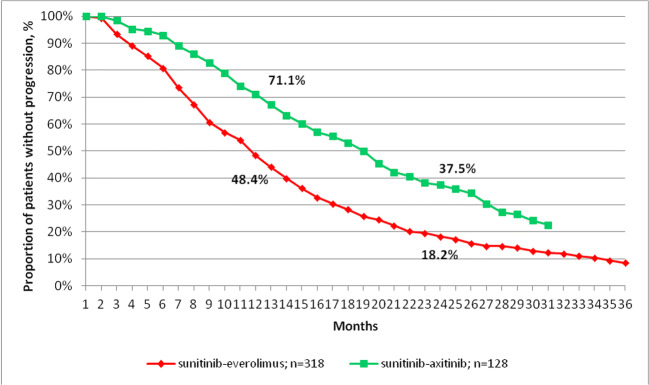
Duration of first-line treatment among patients with mRCC receiving sunitinib-everolimus or sunitinib-axitinib sequential treatment

The DFT of mRCC patients is shown on Fig. 2. Median DFT was significantly longer among patients treated with sunitinib-axitinib compared to those receiving the sunitinib-everolimus sequence (median DFT: 19 vs. 11.7 months; HR: 0.53 [0.42–0.67]; p < 0.0001).

**Fig. 3:**
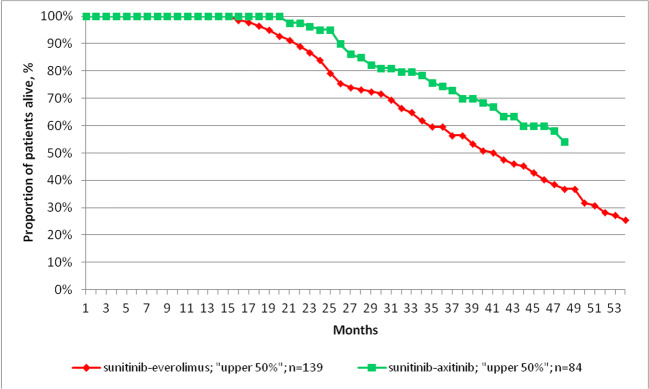
Overall survival among patients with better response to first-line sunitinib therapy who received the sunitinib-everolimus or sunitinib-axitinib sequence

In order to adjust for potential differences in baseline prognostic features of the disease between patient groups, we created patient populations with different response to sunitinib from the pooled population of the sunitinib-everolimus and sunitinib-axitinib patient groups. Using DFT, as described previously both the “upper 50%” (sunitinib-everolimus: n = 139; sunitinib-axitinib: n = 84) and “lower 50%” (sunitinib-axitinib: n = 44; sunitinib-everolimus: n = 179) cohorts included 223 patients, with the median DFT being 14 months in the pooled sunitinib population. Figure 3 shows the OS of patients with better response to sunitinib defined as longer DFT, i.e. the “upper 50%. In this cohort, patients receiving sunitinib-axitinib sequential treatment showed significantly longer median OS than those treated with the sunitinib-everolimus sequence (median OS: 52.8 vs. 41.1 months; p = 0.0019; HR: 0.57 [0.38–0.83]). There was no statistically significant difference in OS with sunitinib-axitinib among patients with poorer response to sunitinib, i.e. the “lower 50%”, although there was a numerical trend towards longer OS with sunitinib-axitinib (median OS: 18.1 vs. 16.0 months; HR: 0.86 [0.56–1.29]; p = 0.462).

Since we found significantly better OS among patients treated with second-line axitinib after sunitinib compared to second-line everolimus, we examined the hypothetical survival gain in years that the sunitinib-axitinib sequence could have provided to “upper 50%” patients who received second-line everolimus therapy (n = 139). The total number of years survived in this patient group since the initiation of first-line sunitinib was 475. Our analysis showed that the administration of the sunitinib-axitinib sequence instead of sunitinib-everolimus would have resulted in an additional survival gain of + 85 years among these 139 patients. Therefore, treating all patients who responded better to sunitinib based on DFT (“upper 50%”) with sunitinib-axitinib would have provided significant survival benefits for this patient group.

## Discussion

This retrospective database analysis investigated the impact of sequences on OS of mRCC patients during the period between January 1 2008 and May 31, 2017. Patients with mRCC receiving sunitinib-axitinib sequential therapy had significantly longer OS compared to those treated with the sunitinib-everolimus sequence and resulted survival gain. However, it was not known whether patients receiving different sequential treatments had different disease prognosis at baseline, which may have influenced overall study results.

One possible explanation for better OS associated with second-line axitinib vs. everolimus is that patients receiving the sunitinib-axitinib sequence had better prognosis at baseline compared to those treated with sunitinib-everolimus. Our findings related to DFT support this assumption as rapid progression on first-line therapy could reflect baseline prognostic features. Indeed, earlier progression could be seen among patients treated with the sunitinib-everolimus sequence (Fig. 2), suggesting that they might have had worse prognosis at baseline, than those treated with sunitinib-axitinib. These patient population may represent the bad responders to first line sunitinib therapy.

To allow for the comparability of OS with different treatment sequences, we stratified patients receiving sunitinib-axitinib or sunitinib-everolimus according to DFT. The OS benefit of the sunitinib-axitinib sequence remained consistent in patient cohorts with similar response to first-line sunitinib therapy. To our knowledge, this is the first published analysis which reports OS data in a real-world population of mRCC patients receiving targeted sequential treatments using the Hungarian NHIF database.

The development of VEGF and VEGFR TKI inhibitors revolutionized the treatment of mRCC. Currently, the most commonly used agents are the tyrosine kinase inhibitors sunitinib and pazopanib, for the first-line treatment of mRCC patients with good or intermediate prognosis [7] stratified by MSKCC risk criteria based on phase III clinical trials [16, 17]. The effectiveness and tolerability of both agents have been confirmed by real-world studies [18, 19]. Everolimus and axitinib emerged as safe and effective second-line treatment options for mRCC patients after progression on VEGF-targeted therapy [14, 20]. However, currently there is no clear guidance on the optimal choice of agents in the second and additional lines of therapy in mRCC, and evidence comparing different sequential regimens is limited to retrospective observational studies [21–24].

Since the present analysis, significant advances have been made in the landscape of first- and second-line therapy in mRCC.

The randomized Phase III CheckMate 214 trial demonstrated that combination therapy with nivolumab and ipilimumab improved OS compared with sunitinib and received regulatory approvel for treatment naive patients with IMDC intermediate and poor risk disease in 2018. Cabozantinib can also be used as first-line therapy due to the results of CABOSUN phase II trial [25] in mRCC of intermediate or high risk group. The phase III JAVELIN trial of avelumab plus axitinib also recently reported improved PFS in this patient’s population compared with sunitinib [26]. KEYNOTE-426 with pembrolizumab and axitinib combination reported improved OS and PFS outcomes versus sunitinib monotherapy in first-line setting [27]. The phase II IMotion 150 trial showed also promising PFS and ORR with combination of atezolizumab and bevacizumab compared with sunitinib, but the OS was not better [28]. Concerning the new data sunitinib is indicated in patients with good risk disease in countries where new innovative treatment is available. In other countries the standard of treatment remains sunitinib as first-line of choice in good and intermediate risk group [29–32].

In second-line therapy cabozantinib showed significantly improved OS, objective response rate (ORR), and PFS compared to everolimus in the large, phase III METEOR trial [33], and was subsequently approved for second-line treatment after prior anti-VEGF therapy among patients with advanced RCC. Lenvatinib, a multi-TKI of VEGFR1–3, fibroblast growth factor (FGF) receptors 1–4, PDGFRα, RET, and KIT also gained approval after showing improved ORR, PFS, and OS in combination with everolimus compared to everolimus alone in a phase II randomized study [34]. The phase III CheckMate-025 study with nivolumab showed significant improvements in OS and ORR when compared to everolimus among patients with previously treated advanced RCC [35]. Based on these findings, nivolumab was the first immune checkpoint inhibitor to be approved in this setting [36].

At the time of our analysis, the first-line therapy consisted of treatment with sunitinib or pazopanib, neither nivolumab nor cabozantinib were available as second-line treatment. Our analysis focused on differences in OS among mRCC patients treated with the sunitinib-everolimus or sunitinib-axitinib sequences.

Previous real-world studies comparing the effectiveness of second-line targeted therapies for mRCC used different designs and reported heterogeneous results, with no convincing evidence of a difference in effectiveness between mTORi and VEGF TKI in the second-line setting [37]. Our results build on the existing real-world evidence which supports the benefits of second-line axitinib treatment over everolimus after prior anti-VEGF therapy [38–40] in patients showing longer DFT on first-line sunitinib treatment.

The main strength of our study lies in the characteristics of the NHIF database which contains all reimbursed medications from all Hungarian mRCC patients receiving targeted therapy over a nearly 9,5-year period.

This study has several limitations which need to be considered when interpreting the results. First, the NHIF prescription claims database does not include any information on patient characteristics, laboratory data, vital signs, baseline prognostic features. A propensity-score based method would have allowed for baseline covariate adjustment, but such approaches could not be applied due to the lack of information of patient characteristics and laboratory data. The adjustment of patient cohorts based on DFT was meant to at least partially eliminate this confounding. Second, in the present study, only effectiveness was evaluated with no assessment of tolerability, which means that we do not have any reliable information about treatment discontinuation and dose reductions.

It is important to emphasize that the decision-making process during the selection of sequential therapy should always be tailored to the individual patient and should involve the careful consideration of patient-related factors including age, prognosis of malignant disease, comorbidities, concomitant medications, compliance as well as potential side effects of therapy. As new evidence is rapidly emerging, it is crucial to discover reliable biomarkers that may help determine the best treatment option for the right patient [41].

## Conclusions

In a real-world population of patients with metastatic renal cell carcinoma, sunitinib-axitinib sequential therapy was associated with improved OS and PFS than the sunitinib-everolimus sequence. Further studies are needed to identify molecular biomarkers that may guide the optimal sequencing of agents and allow for a tailored approach in this patient population.
